# Gender-Informed Family Planning Perceptions and Decision-Making in Rural Chiapas, Mexico: A Mixed-Methods Study

**DOI:** 10.1155/2020/1929143

**Published:** 2020-01-29

**Authors:** Samantha Truong, Jimena Villar de Onis, Alexa Lindley, Rodrigo Bazúa, Andrea Reyes, Mariana Montaño, Leanne Marcotrigiano, Rose L. Molina

**Affiliations:** ^1^Harvard Medical School, 25 Shattuck St., 02115 Boston, MA, USA; ^2^Geneva Foundation for Medical Education and Research, 150 Route de Ferney, 1211 Geneva, Switzerland; ^3^Department of Family Medicine, University of Washington, Box 356390, 98195 Seattle, WA, USA; ^4^Compañeros En Salud, Calle Primera Poniente Sur #25, 30370 Ángel Albino Corzo, Chiapas, Mexico; ^5^Programa Multicéntrico de Residencias Médicas, Tec Salud, Tecnológico de Monterrey, Av Morones Prieto No. 3000, Colonia Los Doctores, CP 64710 Monterrey, Nuevo León, Mexico; ^6^La Clínica de la Raza, P.O. Box 22210, 94623 Oakland, CA, USA; ^7^Department of Obstetrics and Gynecology, Beth Israel Deaconess Medical Center, 330 Brookline Ave, Kirstein 3, 02215 Boston, MA, USA; ^8^Division of Women's Health, Brigham and Women's Hospital, 75 Francis St., 02215 Boston, MA, USA

## Abstract

Compared to other Mexican states, Chiapas possessed the lowest rate of contraception use among women 15−49 years old (44.6%) in 2018. This convergent mixed-methods study assessed family planning use, perceptions, and decision-making processes among women and men in rural communities where Compañeros En Salud (CES) works in Chiapas, Mexico. We conducted surveys of reproductive-aged women and semi-structured interviews with reproductive-aged women, men, and physicians completing their social-service year in CES communities from 2016 to 2017. Of the 625 survey respondents, 368 (58.9%) reported using contraception. The most common methods were female sterilization (27.7%), bimonthly injection (10.9%), and the implant (10.9%). Interviews were completed with 27 women, 24 men, and 5 physicians and analyzed through an inductive approach. Common reasons for contraception use were preventing pregnancy, lack of resources for additional children, and birth spacing. Adverse effects, influence of male partners, and perceived lack of need emerged as reasons for non-use. Male partners often made the final decision about contraceptive use, while women often chose what method. Physicians reported adverse effects, misconceptions about methods, and lack of women's autonomy as barriers to contraception use. Given misconceptions about contraception methods and the dominant role of men in contraception decision-making, our study illustrates the importance of effective counseling and equitable gender dynamics for family planning programming in rural Chiapas.

## 1. Introduction

Reproductive health and rights are necessary to advance women's health around the world by allowing women to prevent unintended pregnancy and avert the morbidity and mortality associated with pregnancy, childbirth complications, and unsafe abortions [1–3]. Mexico has undertaken various efforts to advance access to family planning methods, including the incorporation of family planning into prenatal care guidelines [4, 5], access to free contraception at government clinics, and initiatives to reduce adolescent pregnancies [6]. These programs have resulted in a decrease in the national fertility rate from 6.83 in 1970 to 2.18 in 2016 [7].

Family planning utilization, however, varies by socioeconomic status, delivery location, area of residence, and age [8–11]. The 2009 National Demographic Survey demonstrated a continued disparity in unmet family planning needs between rural (15.9%) and urban (8.1%) areas [12]. A similar disparity in unmet need for family planning persists when comparing indigenous (21.5%) to non-indigenous (9%) women [13]. Other studies have found gender-specific differences in perceived barriers to care, with women more often reporting medical concerns, stigma, and socioeconomic status as factors impeding utilization of contraception than men [14].

As one of the poorest of Mexico's 32 states with 75% of the population living in poverty, Chiapas possesses the nation's highest total fertility rate at 2.89 [15] and highest maternal mortality rate at 68.5 maternal deaths per 100,000 live births [16]. Chiapas also has the lowest rate of contraception use among women 15−49 years old (44.6%) [17]. Compared to the approximately 10.5% of women nationally [18], 50.2% of women in Chiapas who desire family planning are not using any form of modern contraception [19]. Despite substantial quantitative data about contraceptive use and factors associated with non-use, few qualitative studies explore barriers to family planning in Chiapas [20, 21]. While these studies reinforce the overall need to address persistent structural factors that limit family planning access and utilization, there remains a gap in understanding of family planning perceptions and practices, specifically in marginalized, rural non-indigenous communities in Chiapas.

The aim of this study was to quantify family planning use and further explore knowledge and perceptions of family planning among both women and men living in rural communities affiliated with Compañeros En Salud (CES) in the Sierra Madre Mountains. The interviews focused on reasons for use, non-use, or discontinuation of family planning methods, decision-making processes among partners, and perceived positive and negative aspects of contraceptive methods. We also examined the perspectives of physicians regarding knowledge and use of family planning in the CES-affiliated communities where they work.

## 2. Materials and Methods

Compañeros En Salud—the Partners In Health affiliate organization in Chiapas—was founded in 2011 as a healthcare strengthening organization that collaborates with the Mexican government to deliver high-quality medical care through sustainable, community-based engagement and accompaniment in the health system. CES staffs public clinics in 10 rural communities in the Sierra Madre region of southern Chiapas with primary care physicians doing their mandatory year of social service. The population of each community ranges from approximately 600−2100 inhabitants, not including their respective catchment areas.

Unlike other regions of Chiapas, none of the communities affiliated with CES have been identified as indigenous towns by the Chiapas government. At the time of this study, family planning services offered in CES-affiliated clinics included counseling and free access to the following methods: injections, implants, intrauterine devices (IUDs), oral contraceptive pills (OCPs), and condoms. CES organized surgical campaigns for bilateral tubal ligation at least once per year.

We conducted a convergent mixed-methods study to quantify prevalence of family planning methods and understand related perceptions among women and men in CES-affiliated communities. We reviewed a retrospective cohort of two annual “active case finding” surveys conducted in 7 communities and catchment areas served by 4 clinics affiliated with CES in 2016 and 2017 to assess family planning use. In “active case finding” surveys, data collectors visited each house in the community in an attempt to search for all women of reproductive age instead of waiting for them to seek care in the health center. The active case finding surveys occur biannually and are core components of the CES monitoring and evaluation program. Survey data included demographic information, current family planning method, and reasons for non-use. Data collectors were trained in standardized data collection methods. The surveys were administered through a mobile application, CommCare (Dimagi, Cambridge, MA, USA), to all women between 16 and 50 years of age in December 2016 in two communities and one catchment area, and then in July 2017 in four additional catchment areas. The data was exported from CommCare and analyzed in Excel. Continuous variables were presented as medians with interquartile ranges and frequencies were calculated for categorical variables. We excluded pregnant women because they were assumed to not be actively using a form of contraception.

We then conducted qualitative interviews to explore knowledge and perceptions about family planning methods among reproductive-aged women and men and CES physicians between September 2016 and March 2017. The inclusion criteria for participants were: women of reproductive age (15−45 years old), men whose partners were women of reproductive age, and residence in one of the eight CES-affiliated communities. We selected participants through purposeful sampling to ensure variability according to age, gender, parity, neighborhood of residence, and time since last pregnancy. We also sampled from multiple communities to obtain diversity regarding gender of the CES physician working in the community, proximity of the community to a larger town, and different community characteristics (such as income levels, religion, and population size).

Two coinvestigators approached potential participants through home visits, and all household members were screened for the inclusion criteria. One co-investigator is a female, native Spanish-speaker with a law degree and masters in global health. The other co-investigator is a male, Mexican physician and staff member at CES who had previously completed his year of social service in one of the communities. Recruitment continued until 5–8 participants per community, approximately half women and half men, had provided verbal consent and participated in the interview. Each male participant reported on family planning use and decision-making processes with his current female partner. The semi-structured interview guide included questions regarding past and current contraception, perceptions of contraception methods, and decision-making around family planning. We reached saturation when interviews revealed a redundancy of themes.

We conducted semi-structured interviews with physicians at the end of their year of social service with CES to triangulate perceptions of family planning and elicit their perspectives of patient experiences, choices, and factors that influence family planning and decision-making that may not have been captured in community participant interviews alone. Written consent was obtained from physician participants. All physician interviews were conducted in a private room in the CES office.

All interviews were recorded and conducted in Spanish. One coinvestigator and a professional transcriptionist transcribed the interview audio files. All data were de-identified upon transcription of the audio recordings. We analyzed the transcripts using Dedoose (Dedoose Version 8.1.19, web application for managing, analyzing, and presenting qualitative and mixed method research data (2019). Los Angeles, CA: SocioCultural Research Consultants, LLC www.dedoose.com), a qualitative data analysis program. Three study investigators coded the transcripts and created a codebook through an iterative inductive process using grounded theory. All transcripts were cross-coded by two coinvestigators. We compared codes according to gender of the respondent to identify differences in responses and saturation of themes. Excerpts assigned different codes were discussed and the differences were resolved by clarifying code definitions until codes were applied concordantly.

This study received approval from the Bioethics Commission of the Ministry of Health in Chiapas and the Institutional Review Board of Partners Healthcare, Boston, MA.

## 3. Results

There were a total of 625 reproductive-aged women surveyed through active case finding, 441 in 2016 and 184 in 2017.

### 3.1. Prevalence of Contraception Use and Reasons for Non-use in CES Communities

Among the total number of participants, 368 (58.9%) reported using a contraceptive method. The most commonly reported methods of current contraception were female sterilization (173/625, 27.7%), bimonthly injection (68/625, 10.9%), and implant (68/625, 10.9%) (Table 1). Among the 254 women who reported not using a modern contraceptive method, the most commonly reported reasons for not using contraception were: declined family planning method when offered (63/254, 24.9%), sexual inactivity (35/254, 13.8%), lack of a nearby health center (30/254, 11.9%), using a natural family planning method (28/254, 11.0%), and side effects (24/254, 9.5%) (Table 2).

### 3.2. Qualitative Interview Participant Characteristics and Family Planning Utilization

There were 51 participants (27 women and 24 men) in the qualitative interviews. The female coinvestigator interviewed all female participants (*n* = 27) and 63% of the male participants (*n* = 15). The male coinvestigator interviewed the remaining male participants (*n* = 9). Characteristics of the 51 participants from eight CES-affiliated communities are summarized in Table 3. Participants ranged from ages 19 to 56 years old. Parity varied from 0 to 8 children, with an average of 3 children per participant. Most participants were either using a contraceptive method currently or had in the past (39/51, 76.5%). Common types of contraception were injection, implant, IUD, and bilateral tubal ligation. Of the 12 out of 51 (23.5%) participants who had never used any form of modern contraception, 5 (9.8%) had used a natural family planning method, such as withdrawal or rhythm method to prevent pregnancy.

### 3.3. Reasons for Using Contraception

Overall, the main reasons participants used family planning were to prevent pregnancy, the lack of resources to sustain a larger family, and the importance of birth spacing. One participant elaborates on the difficulty of having many children: “*To take care of [my children], to educate them, to dress them, you need all these means, and here we are people with little resources. We don't have money…here it's more difficult…because here there's no fixed work. We live off nothing more than corn, beans.*”Several also commented on avoiding the health risks associated with pregnancy: “*As they tell us in the clinic, having another child, it's a pregnancy that carries its risks. It's the reason why we don't want to have any more.*”Generally, similar themes emerged among both women and men. Women more commonly cited preventing pregnancy and birth spacing while men focused more on lack of resources and health benefits to the mother or father.

Although participants tended to be informed about sexually transmitted infections (STIs), STI prevention was not a salient reason to use contraception generally or condoms specifically, despite most participants' knowledge that condoms prevent STI transmission. The prevalent belief was that within marriage, partners should not have sexual relationships with others and therefore there was no need for condoms. Even when the participant believed that there might be a possibility of infidelity, condom use remained low.

### 3.4. Reasons for Not Using Contraception

The main reasons interviewed participants did not use or discontinued family planning methods were: adverse reactions or complications (whether a known secondary effect or an accepted myth), gender dynamics and influence of male partners, perceived lack of need for family planning, preference for natural family planning methods, or sexual inactivity. Women mentioned negative experiences using contraception that discouraged continued use: “*Well I took [the IUD] out because I wasn't doing well, it felt bad here, it hurt […] I had an infection.*”

Others shared that their desire to use contraception conflicted with their partners' wishes: “*Because he was jealous…therefore he thought that if we started using contraception, I would start sleeping with others.*”

Participants also mentioned general discomfort with the idea of family planning: “*Yes I would tell her [doctor] that she was right [about using family planning], but I don't know, I just never liked the idea of it.*”Others shared that the frequency of taking a method was undesirable. For example, one woman shared “*contraceptive methods are good, they're effective… but you have to have good control. There are people who use a method but we forget. We have our date […] and we forget.*” Finally, some participants mentioned that their preferred family planning method was not available.

### 3.5. Sources and Perceptions of Contraception Counseling

Although participants reported obtaining information from the clinic, government-sponsored education campaigns, and their social networks, many still reported feeling they lacked information about family planning methods. Some participants reported that because they received information about methods through other community members they did not feel the need to discuss contraception with the physician. Participants often judged contraceptive methods based on the practicality of the method, particularly the frequency of administration and adverse effects learned from medical personnel and from stories perpetuated in the community.

Most participants reported positive perceptions of family planning counseling, particularly around feeling that conversations were confidential and that medical personnel treated them with respect. One participant commented, “*Well, in fact, there are times, when...it has to be confidential. Only with the doctor…Yes because you can't go around telling other people, because then people start to whisper and that is not good.*”Another reflected upon satisfaction with care received:“*Ah yes, they [doctors] do it with a lot of respect. They treat us, well the times I have been [to the clinic], they have treated us well.*”Participants also highlighted the benefits of information gained and doubts resolved after family planning counseling, including the following comment: “*People don't know very much. We're a little ignorant, but now to know how many options there are to take care of ourselves, our minds are more open.*”

Negative perceptions surrounding family planning counseling included discomfort and shame while discussing this sensitive topic, perceptions of being reprimanded by medical staff, and feeling unprepared to make decisions about family planning. One participant mentioned discomfort with family planning discussions: “*Well, in reality, it's a little uncomfortable, it's a little embarrassing, but they [doctors] have to ask us the [family planning] questions, because in fact it is for our good.*”Another reports feeling judged by health care personnel: “*Yes, in fact, there were some of the nurses that were reprimanding us, that we have to use a method, that we were having too many children […] in fact there are doctors and nurses that yes, they scold us.*” Others felt like they lacked proper information to decide between family planning methods: “*[I'd like to know more] about what you mentioned, about the IUDs, what reactions they cause, and if it is effective or not, but no, I'm not very well-informed about that.*”

### 3.6. Contraception Decision-Making

Decision-making about contraception centered on conversations between the couple, with the man often making the final decision about whether the couple will use a contraceptive method and the woman making the final decision about what type of method. The most common decision-making processes were couples making the decision about family planning use together, the male partner making the decision, or a woman initiating the conversation and male partner approving her decision.

One woman commented on the importance of joint decision-making: “*I can't just decide I'll go on my own without my husband's consent…this should be a discussion between partners. If there isn't trust, there isn't love. I can't just do what I want. I have to talk with my husband if he agrees or doesn't agree. It should be as such: a discussion between the two of us.*” A male partner similarly emphasized the importance of joint decision-making: “*We're like this: we decide one thing and I go with her, or we decide one thing and she goes with me…together.*”

Participants reported less often that a woman would make the final choice regarding contraception use. In some cases, women were openly able to decide for themselves, while in other cases they used family planning in secret: “*He didn't want me to use contraception because he didn't like it…so that's how I used family planning, hidden, he didn't know.*” A few participants mentioned involvement of the mother-in-law in the decision. Men commonly reported deciding about family planning as a couple. However, women more commonly reported that men would make the final decision, followed by a decision made as partners.

Regarding the decision about which contraceptive method to use, participants most commonly reported making the decision together or the woman provided a preference and her partner approved the decision. Contrary to the decision of whether or not to use family planning, men were less involved in deciding the specific type of contraceptive method to use. Other less commonly reported decision-making processes included female or male partner independently deciding, the male partner explicitly stating it should be the woman's choice (“*He said it's up to me to decide, since I am the one who will suffer the pain of childbirth*” or “*My partner told me to choose whatever method is best for me*”), deferring to medical personnel, or the male partner stating a preference and the woman then approving it. Men most commonly reported making the decision as partners, while women reported voicing the choice before seeking approval of their partners.

Although participants referenced decision-making as a couple, more in-depth probing revealed that this was often the male partner approving of the woman's decision. While in some circumstances, men agreed with their partners (“*I already had six children so I told my husband they were going to operate on me…and he said it was okay*”), several participants shared that the male partner held the final decision (*“Some men are *machista* and they say the woman is going to have their children if necessary” or “I got, I think, the implant, but my husband then didn't want it anymore, apparently it's bad, so then no, I didn't want to continue.”*)Overall, few participants mentioned that women alone could decide about family planning use or type of method.

### 3.7. Physician Perspectives of Community Members' Family Planning Perceptions and Practices

Five physicians (3 women and 2 men) were selected for interviews. Three of the five physicians worked in the same communities represented in the community participant interviews. Although two physicians worked in a CES-affiliated community not represented in the community participant interviews, themes that emerged from these interviews aligned with the others.

In general, physicians felt patients were receptive to hearing about family planning options. However, several barriers prevented family planning use, including adverse effects, misconceptions or lack of knowledge about family planning methods, gender dynamics, lack of women's autonomy in family planning choices, religious beliefs, and stigma surrounding contraception. One physician described challenges with adverse effects of contraceptive methods: “*My primary barrier as of late has been that women don't return to the clinic, that we give them the bimonthly injection one time, they start bleeding, and although you explain to them that this is normal, they don't come back.*”While religion did not emerge as a theme in the other interviews, several physicians perceived that family planning use varied by religion. For example, one commented “*it depends a lot on religion, for example, the Pentecostals are the most close-minded, or the most private about this, above all the men.*”

Women often came to clinic with preconceptions from other community members about adverse effects and which method to use. Common misconceptions included the fear that certain methods would cause cancer or methods that halted menstruation would cause blood to harmfully accumulate in a woman's uterus: One physician noted: “*It's pure fear and lack of knowledge. I had a period when people were saying that the implant caused cancer and that it made you sick and it would travel through your body and all of this misinformation is a gigantic barrier; that many of the prejudices can end if, before offering a method, you give an extensive overview and you take time to explain to each woman how the methods work. For example, there are methods that make your menstrual cycle irregular or even make it disappear completely; this worries many women, sometimes they ask where that blood is going.*”Another physician highlights the influence of community norms on contraceptive choices: “*I've taken out other [methods] because everyone makes the woman scared, because she comes and tells you that for these reasons she no longer wants it, that she already feels bad, but really people have the most influence over her and multiple women have told me that.*”

Physicians expressed frustration with women's lack of agency, commenting on the significant influence of men and mothers-in-law over family planning decisions: “*It's terrible that they depend so much on the husband. It makes me incredibly stressed to talk with them and to see that women want [family planning] and that they're fed up, that they don't want any more children, and that they can't make that decision.*”While some physicians observed that men let their partners decide which method to use, others perceived that men still made the final decision regarding whether to use family planning. In situations with conflicting opinions between couples regarding family planning, physicians attended to women's choices instead of their partners' preferences.

Other themes that emerged regarding gender dynamics included (1) the belief that family planning would enable infidelity and (2) the observation that men did not accompany their partners in clinic visits or government-sponsored health educational talks despite their influential role in family planning decision-making. While physicians occasionally conducted family planning consults with couples, the majority of these consults were done with women alone. Physicians believed that STI prevention had minimal impact on decision-making surrounding family planning, which concurred with what was found in the community participant interviews.

## 4. Discussion

This mixed-methods study combines CES monitoring and evaluation surveys and qualitative interviews with reproductive-age women and men, and community physicians to provide a gender-informed landscape of family planning use, perceptions, and decision-making in a rural region in Chiapas, Mexico. While previous studies have been conducted in rural indigenous and urban non-indigenous communities, our population was drawn from rural non-indigenous communities where family planning practices have been minimally studied. While multiple studies examine the disparities in family planning between indigenous and non-indigenous populations, this study offers an exploration in rural, low-income communities where gaps persist in access to and utilization of contraceptive methods [8, 12, 22]. A more nuanced understanding of these populations will facilitate family planning programs to more specifically meet the needs of women in such regions. Aside from community health worker trainings about basic family planning methods, no CES-related interventions related to family planning were happening during the time of the study.

According to the survey results, nearly half of women were not using any form of contraception, a notably higher proportion than the 27.4% of women nationally [18], highlighting continued disparities between rural and urban areas. The most common form of contraception, by nearly triple, was bilateral tubal ligation and hysterectomy, which reflects trends in high rates of female sterilization in Latin America, and particularly at earlier ages in Mexican women [23–25]. Extensive campaigns for bilateral tubal ligations and continued distrust of hormonal methods likely contribute to high rates of female sterilization [20]. Many participants mentioned limited economic resources as a reason for preventing pregnancy, which has been shown to be a primary reason for female sterilization in the border region of Chiapas [21].

In assessing reasons for non-use, we found that some respondents expressed a general lack of desire for contraception (lack of interest, abstinence, or no perceived need for family planning), while others expressed openness to family planning but cited barriers (adverse effects, gender dynamics, no nearby health center, or preference for natural family planning methods). Despite physician perspectives about the influence of religious beliefs (mentioned by four out of five participants as a barrier to family planning), no participants in our surveys and only one participant in our interviews mentioned the influence of religious beliefs on family planning preferences. These findings contrast to those of a prior qualitative study in rural, indigenous and urban, non-indigenous municipalities in Chiapas, which identified strong religious objections to contraception in a focus group of indigenous men from rural communities, suggesting that religion may be less of a barrier than prior studies have indicated [20].

Our findings corroborate those of other studies in Mexico: that perceived adverse effects—some of which were evidence-based and some that were not— are primary factors that influence decision-making surrounding family planning methods [11, 20]. Together, these studies highlight the continued need for improved family planning counseling. Given that several participants felt that they lacked information about family planning methods, patient-centered counseling is particularly crucial given the extensive history of procedures without informed consent in vulnerable populations [26]. Our findings specifically demonstrate the importance of tailoring family planning counseling to dispel myths and adequately prepare women for the potential side effects of each method [11, 20].

Some participants reported that decision-making surrounding family planning involved discussions between the couple, though when further probed, many participants revealed that male partners carried a more influential role in the decision than women. Our results contribute to the significant body of literature supporting the influential role of gender dynamics in decision-making processes among couples [27, 28]. For example, Kolodin et al. [29] describe how in certain regions of Chiapas and Guatemala, male partners are the primary decision-makers between the couple, often with great influence from their mothers, as most women tend to live with the husband's family after marriage. Our interviews support similar conclusions from a qualitative study in a separate set of municipalities in Chiapas, which reported that while family planning was often considered to be a decision made between partners, power dynamics emerged in which men dominated health-related choices [20, 28]. However, similar to a qualitative study by Dansereau et al. [20], our interviews reveal a wide spectrum regarding the influence of male partners, with some partners leaving the contraceptive decision-making regarding solely up to women. Although men wielded significant influence over the decision-making process surrounding family planning, few participants reported that men attended government-sponsored health educational talks or clinic visits with their partners. Dansereau et al. [20] also found a lack of involvement of male partners in intervention strategies. Our findings suggest that actively including men in program interventions is key to successfully promoting contraceptive methods in the communities [30–33].

Supply availability of various family planning methods was reported as inconsistent, and some methods that community members favored, in particular trimensual injections and implant, tend to be more expensive and more frequently unavailable. Similarly, Dansereau et al. [20] found that women reported common stock-outs of the implant and injections. These findings highlight the importance of addressing supply-side barriers to family planning utilization.

Participants discussed the importance of trust and confidentiality, feeling respected, and receiving understandable information about family planning, similar to another study of women's preferences for contraceptive counseling in Mexico [34]. However, some participants reported experiences of discomfort while discussing contraception and being reprimanded by medical personnel, which point to a persistent challenge in delivering nonjudgmental and respectful patient-centered counseling.

## 5. Limitations

Given the exploratory nature of this study, there may be limited generalizability to other rural communities in the region. The responses in the quantitative surveys were limited by incomplete categorization of fertility desires. While women were asked about family planning utilization, we did not distinguish between women who hoped to become pregnant and those seeking to prevent pregnancy.

Purposeful sampling of interview participants allowed for a diversity of perspectives but may not be fully representative of all community members. Given the sensitive nature of family planning, participants may have responded differently to questions depending on the gender of the interviewer. However, even when we had gender concordance between interviewer and respondent, we did not identify any differences in the responses based on gender. Additionally, our findings are subject to social desirability bias given that CES-affiliated staff members conducted the interviews. While we trained the interviewers to exercise reflexive practices to monitor their own biases during the interviews, we recognize that participants may have responded in ways to acquiesce to the interviewer. However, we did find variation in responses, some of which were critical of the counseling received, which point to participants' ability to answer questions candidly. Lastly, our findings are limited to perceptions and decision-making preferences comparing men and women, though we recognize that gender dynamics often shape lived experiences and are difficult to capture through semi-structured interviews. We did not ask about other factors that have been shown to influence women's decision-making power, such as education level or control over financial resources [27].

## 6. Conclusion

Our study illustrates the importance of effective contraceptive counseling and gender dynamics in decision-making for family planning programming in rural Chiapas. Further research could explore factors that influence women's decision-making power surrounding family planning and their expectations for family planning counseling in rural regions of Chiapas. As a result of this study, the CES maternal health program is working with community health workers and clinic staff to improve family planning counseling, including developing new ways to communicate possible secondary effects and dissipate existing myths surrounding methods. The program is also increasing efforts to involve men in family planning education and interventions. Bridging the information gaps among women, men, and their physicians will contribute to improved patient-centered counseling and better meet the family planning needs of women in rural and impoverished regions of Mexico.

## Figures and Tables

**Table 1:** Modern contraceptive use among surveyed women aged 16 to 50 in 2016−2017.

Contraceptive method	Reproductive-age women (*n* = 625)
No method	254 (40.6%)
Tubal ligation or Hysterectomy	173 (27.7%)
Bimonthly injection	68 (10.9%)
Implant	68 (10.9%)
Condoms	23 (3.7%)
Monthly injection	18 (2.9%)
Intrauterine device	9 (1.4%)
Oral contraceptive pills	9 (1.4%)
Declined to respond	3 (0.5%)

**Table 2:** Primary reason for not using modern contraceptive methods among women aged 16–50 not using a method in 2016−2017.

Reason	Reproductive-aged women not using a modern contraceptive method (*n* = 254)
Not interested	63 (24.9%)
Denies sexual activity	35 (13.8%)
No nearby health center	30 (11.9%)
Uses natural method	28 (11.0%)
Side effects	24 (9.5%)
Not familiar with methods	19 (7.5%)
Menopause	13 (5.1%)
Uncomfortable	8 (3.2%)
Has not been offered	8 (3.2%)
Illness	6 (2.4%)
Nonreligious beliefs	4 (1.6%)
Infertility	4 (1.6%)
Family member's preference	3 (1.2%)
Contraindication	3 (1.2%)
Side effects in infant	2 (0.8%)
Declined to respond	4 (1.6%)

**Table 3:** Characteristics of community participants in qualitative in-depth interviews.

	Female participants (*n* = 27), *n* (%)	Male participants (*n* = 24), *n* (%)
*Age*		
Median (IQR)	29.5 (23.5–34.5)	35.0 (28.5–41.5)
Range (years old)	20–44	19–56

*Relationship status*		
Cohabitation	24 (88.9)	21 (87.5)
Single	3 (11.1)	3 (12.5)

*Parity*		
No children	2 (7.4)	3 (12.5)
1-2 children	9 (33.3)	4 (16.7)
3–5 children	13 (48.1)	13 (54.2)
6 or more children	3 (11.1)	4 (16.7)

*Time since last delivery*		
No previous delivery	2 (7.4)	3 (12.5)
Delivery within past year	6 (22.2)	5 (20.8)
Delivery > 1 year ago	19 (70.4)	16 (66.7)

*Use of contraception * ^∗^		
Currently using modern contraceptive method	14 (51.9)	17 (70.8)
Used modern contraception method in the past but not currently using	5 (18.5)	3 (12.5)
Never used any modern contraceptive method, but used a natural method	3 (11.1)	2 (8.3)
Never used any form of contraception	5 (18.5)	2 (8.3)

*Current contraceptive methods * ^∗∗^		
\No current use	13 (48.1)	7 (29.2)
\Bilateral tubal ligation/hysterectomy	5 (18.5)	6 (25.0)
Withdrawal/rhythm method	5 (18.5)	2 (8.3)
Injection	4 (14.8)	6 (25.0)
Implant	3 (11.1)	2 (8.3)
Condom	1 (3.7)	2 (8.3)
Intrauterine device	1 (3.7)	1 (4.2)
Patch	0 (0.0)	1 (4.2)

*Prior contraceptive method use * ^∗∗^		
Injection	15 (55.6)	4 (16.7)
No past use	6 (22.2)	6 (25.0)
Withdrawal/rhythm method	6 (22.2)	5 (20.8)
Intrauterine device	3 (11.1)	3 (12.5)
Patch	1 (3.7)	0 (0.0)
Oral contraceptive pills	1 (3.7)	1 (4.2)
Condom	1 (3.7)	9 (37.5)

^∗^Male responses reflect family planning practices of current female partner.

^∗∗^Some participants reported using more than one contraceptive method, so total usage in these subsections exceeds the total number of participants.

## Data Availability

The data used to support the findings of this study are not available to the public as restricted by the Bioethics Commission of the Ministry of Health in Chiapas and Institutional Review Board of Partners Healthcare, Boston, MA in order to protect patient privacy. The informed consent to which participants agreed specified that only members of the study team would have access to the data. Descriptive analysis of internal monitoring and evaluation data was performed in Excel, so no code files exist to share.
